# Motivation Relationships with Physical Activity and Resistance Training Engagement, and Health and Fitness of Law Enforcement Officers

**DOI:** 10.3390/healthcare13212701

**Published:** 2025-10-26

**Authors:** Kristine J. Sanchez, Maria M. Beitzel, J. Jay Dawes, Robin M. Orr, Joseph M. Dulla, Robert G. Lockie

**Affiliations:** 1 Tactical Fitness and Nutrition Lab, Oklahoma State University, Stillwater, OK 74078, USA; krissy.sanchez@okstate.edu (K.J.S.); jay.dawes@okstate.edu (J.J.D.); 2 Center for Sport Performance, Department of Kinesiology, California State University, Fullerton, Fullerton, CA 92831, USA; mbeitzel@fullerton.edu; 3 Tactical Research Unit, Bond University, Gold Coast, QLD 4226, Australia; rorr@bond.edu.au (R.M.O.); joseph.dulla@student.bond.edu.au (J.M.D.)

**Keywords:** behavioral regulation in exercise questionnaire, body composition, Godin leisure-time exercise questionnaire, incumbent officers, muscular endurance, police, push-ups, resting heart rate, sit-ups, tactical

## Abstract

**Background/Objectives**: There are generally no mandates for law enforcement officers to maintain career fitness. Evidence documenting the motivation of officers who are physically active could support training and health and wellness initiatives, while preventing disease (e.g., cardiovascular, metabolic) in this population. This cross-sectional study derived relationships between motivation, physical activity (PA), resistance training (RT) participation, and health and fitness in officers. **Methods**: Sixty officers completed a questionnaire assessing PA (weekly strenuous, moderate, mild exercise sessions; activity score) and RT (RT frequency [RTF]; weekly sessions over 3 months [RT3M]; sessions in past 7 days [RT7D]). Motivation (amotivation, external, introjected, identified, integrated, and intrinsic regulation) was measured via the Behavioral Regulation in Exercise Questionnaire. Health and fitness tests included resting heart rate (RHR), blood pressure, skeletal muscle mass (SMM%) and fat mass (FM%) percentage, waist-to-hip ratio, sit-and-reach, grip strength, push-ups, sit-ups, and step test. Spearman’s correlations (*p* < 0.05) derived relationships between motivation and all other variables. **Results**: All intrinsic motivation styles correlated with strenuous exercise sessions, RTF, RT3M, and push-ups (ρ = 0.286–0.670). Identified, integrated, and intrinsic regulation correlated with activity score and sit-ups (ρ = 0.287–0.472). Identified (ρ = 0.444) and integrated (ρ = 0.341) regulation related to RT7D. Amotivation related to RTF (ρ = −0.295) and RT3M (ρ = −0.290). External, introjected, and identified regulation correlated with RHR (ρ = ±0.270–0.338). Integrated and intrinsic regulation positively related to SMM% and negatively related to FM% (ρ = ±0.265–0.323). **Conclusions**: Internally motivated officers completed strenuous exercise and RT, and had better RHR, body composition, and muscular endurance. Training staff should develop intrinsic motivation styles in personnel to enhance their well-being.

## 1. Introduction

A career as a law enforcement officer can be physically demanding [1]. Officers perform various demanding tasks which include, but are not limited to, checking bona fides, driving vehicles at high speeds, climbing flights of stairs, dragging people and objects, engaging in foot pursuits, and apprehension of suspects [2,3]. Multiple components of fitness form the foundation for the physical ability to perform demanding job tasks [2,4,5,6]. Results from two studies by Lockie et al. [4,5] suggest that multiple components of fitness are related to law enforcement job tasks in recruits. To provide some specific examples, a faster 99-yard obstacle course performance significantly related (*r* = ±0.13–0.53) to better muscular strength and endurance (measured by push-ups, sit-ups, pull-ups, and mountain climbers), power (measured by the vertical jump and medicine ball throw), and running tests (measured by a 220-yard run, 1.5-mile run, 75-yard pursuit run, and multistage fitness test shuttles) [4,5]. A faster 165 lb body drag over approximately 11 yards related to better isometric strength measured by grip and leg/back dynamometers (*r* = −0.26 to −0.67) [7], and lower-body power measured by vertical and standing broad jumps (*r* = 0.21–0.61) [8]. Aerobic fitness has also been suggested to underpin many law enforcement tasks, especially when performed in succession [2,6,9]. Accordingly, higher overall fitness should contribute to the efficient performance of law enforcement-specific tasks.

Nonetheless, reductions in fitness among law enforcement officers have been observed over the course of their careers [1,10,11]. As an example, the cardiovascular fitness of an officer tends to decline faster than that of the general population [1]. Frick et al. [1] analyzed the cardiovascular fitness of 83 officers (79 men, 4 women) from one medium-sized police department relative to American College of Sports Medicine (ACSM) guidelines. The results indicated that in comparison to the general population, officers aged 20–29 years were 13.6% below the ACSM guidelines, officers aged 30–39 years were 21.2% below, and officers 40+ years of age were 25% below. Lockie et al. [12] found that law enforcement officers who spent 48 months or more working in custody facilities had approximately 20–24% worse muscular endurance measured by sit-ups completed in 60 s, compared to officers who had worked for less time. Increased age can be a factor as it results in a decrease in physiological characteristics, such as skeletal muscle mass [13], that are directly related to qualities such as speed, strength, and power [14]. However, other physiological and lifestyle factors can influence fitness declines. Shift work, poor stress management and dietary choices, inadequate sleep hygiene, and a lack of physical activity (PA) can all impact an officer’s health and fitness [1,15,16,17]. Reductions in PA could be very impactful in this regard [18,19,20]. Even though aspects of their job are physically demanding [21], officers will experience long periods of sedentary activity when on-duty, such as performing administrative tasks being seated while patrolling in a vehicle [1,22]. Additionally, as officers progress through their careers, their roles may shift to one that is more supervisory in nature, thus increasing sedentary activity [16].

PA has been defined as any bodily movement produced by skeletal muscles that requires energy expenditure [23]. An increase in PA outside of work hours for law enforcement officers could potentially combat the effects of job-related physical and mental stressors [24]. Moreover, participating in regular and intentional exercise, such as anaerobic and aerobic conditioning, and resistance training (RT), could improve a range of fitness traits ultimately beneficial to the job. Specific to law enforcement occupational tasks, appropriate fitness training could improve functional movement, lifting heavy objects, muscular endurance, jumping, and running [25,26,27]. Officers who are physically active could also experience reduced injuries over the course of their career, as well as overall better career longevity [20,25]. However, law enforcement officers are generally not mandated to meet any fitness standards after they finish their training academy [28], which could disincentivize some officers from completing PA, given their job-related barriers (i.e., shift work, disrupted sleep and fatigue, stress). Indeed, these barriers could contribute to a decrease in motivation and/or complete absence of motivation to engage in PA [29,30]. Given the potential value of PA in officers to help cope with stress and to enhance job task performance [2,4,5,7,8,9,24], in conjunction with clear fitness declines experienced by many officers [1,10,11], there is a need to explore the nuances of motivation to be physically active among law enforcement officers.

Self-determination theory has been used to study motivation to exercise [31,32]. The theory is based upon the idea that there are different types of motivation that influence personal behavior [32]. Those who engage in an activity for an inherent sense of satisfaction would do so out of intrinsic motivation, or because they are internally motivated [32,33,34]. Those who participate in an activity, or exercise, for the sake of achieving a specific goal, rather than for the enjoyment of the activity itself, would do so because they are extrinsically or externally motivated [32,34]. As described by Ryan and Deci [31] and Palombi et al. [35], within these types of motivation that are six regulatory styles that illustrate a continuum. Amotivation occurs when a person does not value exercise and has no intention to perform exercise, and is non-self-determined. External regulation occurs when exercise is performed to satisfy external demands (e.g., to obtain a reward or avoid punishment). Introjected regulation is somewhat external and occurs when exercise is regulated by internal rewards to increase pride or self-esteem, or to avoid guilt. Identified regulation is somewhat internal and is when personal importance and value is placed on exercise by the individual. Integrated and intrinsic regulation are internal and occur when the individual is self-determined. When exercise is consistent with one’s identity, values, and needs they have integrated regulation. Intrinsic regulation is when exercise participation occurs due to personal interest, inherent satisfaction and enjoyment. To encourage PA and lifelong fitness in officers, it would be prescient for staff to develop identified and intrinsic regulation during academy.

Despite officers learning necessary occupational skills during their time in the academy, motivation to maintain their physical fitness may not be developed or further fostered post-academy. As previously stated, many law enforcement organizations do not enforce a PA mandate or fitness standards once officers are officially employed [28]. This removes extrinsic motivation to exercise, which is notable as extrinsic motivation is the overwhelming motivational tool used during law enforcement training academies. Training staff use external motivation with recruits via expectations to follow orders, established fitness standards, expectations for training, performance reviews, and avoidance of punishment [36,37,38]. Accordingly, recruits may not receive coaching and education as to why they are completing exercise [37], which could inhibit development of their intrinsic motivation to exercise. The downstream effect of this is lack of motivation to exercise once an officer starts working. If officers are not mandated to maintain a certain level of fitness and given the PA to improve the health and fitness of officers, it is important to investigate potential reasons why some officers remain physically active post-academy while others do not. This is important in the context that there are generally no mandated strategies as it relates to officer motivation and fitness, with mandates typically relating to only the number of hours allocated for fitness training [39], if they exist at all [28].

Therefore, the purpose of this study was to explore the relationships between the motivation of law enforcement officers to engage in PA and/or RT with health and fitness outcomes measures. There is currently no research that has investigated relationships between motivation and PA, RT, and health and fitness in law enforcement officers, so the results from this study will be very novel. Law enforcement officers completed questionnaires regarding the frequency and intensity of PA and RT [40], and exercise motivation [41,42]. The officers then completed a series of health and physical fitness tests [12,27,43]. It was hypothesized that officers who exhibited greater intrinsic motivation would be more physically active and have better health and fitness indicators (e.g., greater lean body mass, decreased resting heart rate, greater muscular strength and endurance, and better aerobic fitness).

## 2. Materials and Methods

### 2.1. Subjects

Archival data from a law enforcement agency in Southern California was analyzed. The sample of convenience of de-identified data included 60 officers (age: 32.08 ± 5.66 years; height: 1.72 ± 0.08 m; body mass: 86.46 ± 16.32 kg; years of service: 4.80 ± 3.47 years), were analyzed. The sample included 48 men (age: 30.87 ± 5.66 years; height: 1.75 ± 0.07 m; body mass: 91.33 ± 14.18 kg; years of service: 4.40 ± 3.30 years) and 12 women (age: 36.83 ± 6.65 years; height: 1.61 ± 0.03 m; body mass: 67.00 ± 7.72 kg; years of service: 6.36 ± 3.81 years). The researchers did not control the final sample size used for the study as this was dependent on the available data provided by the agency. Moreover, data such as that from the current study is often not available due to numerous challenges associated with conducting research with law enforcement personnel [39,44], so larger sample sizes are generally not possible. Data from these officers has also featured in other published research [12,45]. The inclusion criterion for the officers were participating in patrol school and had complete data sets for the variables of interest; officers would be excluded if their data sets were incomplete, but no officer datasets available to use in this sample were excluded. The institutional ethics committee approved the use of pre-existing data (HSR-17-18-370), and the research was conducted according to the Declaration of Helsinki [46].

### 2.2. Procedures

The data were collected by staff working for one agency during patrol school in Spring of 2018. Patrol school was a three-week skills refresher program completed by officers who had been working in custody, as they did not complete any patrol duties during this time [12,45]. Officers wore physical training attire (i.e., no equipment) for all general and job-specific fitness tests. The PA questionnaire, motivation questionnaire, and health and fitness testing was completed voluntarily by officers who opted into the assessment [12]. Testing was conducted indoors on a basketball court at the agency’s training facility in groups of 10–15. Depending on availability, officers were tested in the morning or early afternoon and were not fasted prior to testing. As the officers had other commitments within patrol school related to skill and procedural development [12,45], staff could not be expected to exercise tight constraints on personnel such as that adopted in laboratory testing. Indeed, there is a lack of flexibility in how data is collected in law enforcement populations, although the inherent advantage is the data accurately represents what officers look like in the real world [44]. The questionnaires were completed first independently by the officers, which was then followed by resting heart rate (RHR) and blood pressure (BP) measurement, and then height and body mass. The officers then progressed had their waist and hip measurements taken, and completed the sit-and-reach and hand grip tests. The group completed push-ups and sit-ups together, before completing the YMCA step test.

### 2.3. Godin Leisure-Time Exercise Questionnaire—Physical Activity Questionnaire

The Godin Leisure-Time Exercise Questionnaire assessed physical activity [40], with the questionnaire used in this study displayed in Table 1. This questionnaire considered the average frequency of mild, moderate, and strenuous physical activity over a 7-day period, as well as overall activity score. Activity score was calculated via the formula [40]:

Activity Score = (9 × Number of Strenuous Sessions) + (5 × Number of Moderate Session) + (3 × Number of Light Sessions).

Participation in RT was assessed through another three items addressing how often the officer engaged in RT in the past 3 months (resistance training frequency; RTF), the average number of days RT per week in the past 3 months (RT3M), and the number of sessions in the past 7 days the officer engaged in RT (RT7D).

### 2.4. Behavioral Regulation in Exercise Questionnaire (BREQ-3)—Motivation Questionnaire

Motivation was measured using the 24-item Behavioral Regulation in Exercise Questionnaire (BREQ-3) [41,42]. The BREQ-3 assessed the six types of motivation regulatory styles (in this study, the term ‘amotivation’ was used instead of ‘non-regulatory’), with four items corresponding to each subscale. Each item was scored on a Likert scale from of 0 (not true for me) to 4 (very true for me) (Table 2). There relevant questions for each subscale were:

- Amotivation: 2, 8, 14, 20;
- External Regulation: 6, 12, 18, 24;
- Introjected Regulation: 4, 10, 16, 22;
- Identified Regulation: 1, 7, 13, 19;
- Integrated Regulation: 5, 11, 17, 23;
- Intrinsic Regulation: 3, 9, 15, 21.

The responses to the four questions relevant to the regulatory styles were added together to provide the final score for the relevant motivation styles [47].

### 2.5. Resting Heart Rate (RHR) and Blood Pressure (BP)

RHR and BP were recorded after the officer sat quietly for approximately 5–10 min. Electronic BP monitors (Omron Healthcare, Kyoto, Japan) were utilized due to ease of use, consistency, and need for time management during patrol school [12]. The researchers were not given any information about whether officers were on BP medication, as this was not provided in the dataset. Nonetheless, all the officers had been cleared to participate in the health and fitness testing. Officers sat with their feet flat on the floor and their arm placed in a supported, relaxed position at heart level. Their clothing was repositioned such that the cuff was placed on bare skin without any compression above the cuff. The cuff position was above the crease of the elbow and encircled approximately 75–100% of the arm [12,27,43]. Staff then followed the directions presented on the automated device. RHR (beats per minute; bpm), and systolic and diastolic BP (milliliters of mercury; mmHg), were recorded.

### 2.6. Height, Body Mass, Skeletal Muscle Mass Percentage (SMM%), Fat Mass Percentage (FM%)

Height was measured barefoot using a portable stadiometer (Seca 217, Hamburg, Germany). Body mass, skeletal muscle mass percentage (SMM%), and fat mass percentage (FM%) were recorded by electronic digital scales (Model HBF-510, Omron Healthcare, Kyoto, Japan). Manufacturer guidelines were used to record SMM% and FM% [48]. Age, height in centimeters (cm), and biological sex of the officer were entered into the scale. The officer stepped onto the scale barefoot with their feet positioned on the foot and heel electrodes, and held the display unit electrodes with both hands until their body mass was displayed on the screen. They stood upright and extended their arms so they were parallel to the ground. The scan was completed when the officer’s body mass was displayed again. Proprietary equations provided the measurements of SMM% and FM% [49].

### 2.7. Waist-to-Hip Ratio (WHR)

Waist-to-hip ratio (WHR) measures body fat distribution [50]. Staff used a thin-line metric tape measure (Lufkin, Apex Tool Group, Sparks, Maryland) to determine waist and hip circumfence for all officers [51]. Waist circumference was measured in cm at the narrowest part of the waist. Hip circumference was measured in cm at the greatest posterior protrusion of the hip. WHR was derived by dividing the waist circumference by hip circumference.

### 2.8. Grip Strength

Grip strength provided a metric for upper-body strength [52], and was measured by a hand grip dynamometer (Takei Scientific Instruments, Tokyo, Japan). Officers kept their testing arm by their side while standing and squeezed the dynamometer handle as hard as possible for 2 s [27,43,48,51]. Two attempts were completed for each hand and recorded to the nearest kg, with the left hand tested first. The best score for each hand was added together to provide a combined metric. These grip strength procedures, including the summation of both the left and right hand scores for the final grip strength measurement, were used as they have been commonly adopted across numerous law enforcement populations and studies [27,43,48,51]. Conducting the grip strength procedures in this way allows for greater context and applicability for the measurements.

### 2.9. Sit-and-Reach

The sit-and-reach measured hamstring flexibility [53], using previously documented procedures [54]. The officer removed their shoes and sat with both feet flat against the sit-and-reach box and positioned their hands on top of each other, with the tips of the middle fingers aligned and palms down. The officer then reached forward slowly and touched as far along the scale as possible while keeping the knees extended, and held this position for 5 s. Three trials were performed, with the furthest reach distance used.

### 2.10. Push-Ups

Upper-body muscular endurance was assessed via a 60 s push-up test. The procedures that were adopted mirrored those described by the ACSM [55] and National Strength and Conditioning Association [56]. However, there were modifications used (i.e., methods used to monitor push-up depth) specific to law enforcement populations [12,27,43]. A staff member placed a fist on the floor directly under the chest of the officer to ensure the correct depth. All female officers were partnered with a female staff member. On the start command, the officer flexed their elbows and lowered themselves until their chest contacted the staff member’s fist before they extended their elbows to return to the start position. The officer performed as many push-ups as possible with this technique in 60 s, with the result being the number of correctly completed repetitions.

### 2.11. Sit-Ups

Abdominal muscular endurance was assessed via a 60 s sit-up test. The sit-up test is a common assessment within law enforcement personnel [57], so specific procedures used previously for law enforcement personnel were adopted in this study [12,27,43]. Similarly to the grip strength procedures, conducting the sit-up procedures in this manner allows for greater context and applicability for the results. The officer laid on their back with their knees flexed to 90°, heels flat on the ground, and arms crossed over their chest. Their feet were held in place by a staff member who also counted the repetitions. On the start command, the officer raised their shoulders from the ground while keeping their arms crossed over their chest and touched their elbows to their knees. The officer then descended back down until their shoulder blades contacted the ground. The officer completed as many sit-up repetitions as possible in 60 s with this technique. The recorded result was the number of correctly completed repetitions.

### 2.12. YMCA Step Test

The YMCA step test was administered to measure aerobic capacity via standard procedures [12,27,43]. The test was performed with approximately ~31 cm high bleacher seats used for the step. Officers completed the step test in groups of 6–8 so they could be paired with a staff member to measure their recovery heart rate. To complete the YMCA step test, the officer stepped in time to a 96 bpm metronome continuously for 3 min. The beat was played from an iPad handheld device (Apple Inc., Cupertino, CA, USA) connected to a portable speaker (ION Block Rocker, Cumberland, MD, USA), which was positioned on the bleachers in front of the officers. Following the 3 min time period, the officer immediately sat on the step while recovery heart rate (HR) was manually measured by a staff member via the carotid or radial artery for 60 s.

### 2.13. Statistical Analysis

Statistical analyses were computed using the Statistics Package for Social Sciences (Version 29.0; IBM Corporation, Armonk, NY, USA). Descriptive data (mean ± standard deviation [SD]) were calculated for all variables. Preceding the correlation analysis, normality of the data was evaluated by visual analysis of Q-Q plots and the Kolmogorov–Smirnov test. Comparisons were made between the male in female officers for the motivation subscales, PA, RT, and the health and fitness tests. If the data for a test was normally distributed, independent samples *t*-tests (*p* < 0.05) were used. If data was not normally distributed, then Mann–Whitney U-tests were used for between-sex comparisons. Effect sizes (*d*) were derived for all between-sex comparison, where the difference between the means was divided by the pooled SD [58]. A *d* less than 0.2 was considered a trivial effect; 0.2 to 0.6 a small effect; 0.6 to 1.2 a moderate effect; 1.2 to 2.0 a large effect; 2.0 to 4.0 a very large effect; and 4.0 and above an extremely large effect [59]. Effect sizes were included in this study to ascertain the magnitude of difference between the sexes irrespective of the data distribution and statistical approach adopted, and to provide additional information for the practitioner [60].

As will be detailed in Section 3, the motivation, PA, and RT data was not normally distributed. This meant that Spearman’s correlations (ρ) were utilized to calculate relationships between motivation, PA, RT, and the health and fitness tests, with men and women combined in the analysis. The researchers acknowledge that previous law enforcement research has shown that in general, males as a group tend to outperform females in a range of fitness tests [10,60], which is also reflected in the current results. However, numerous other studies have combined the sexes in their analyses [9,27,48,51,61], and this, in conjunction with the data distribution in the current research, and lack of between-sex differences in motivation, PA, and RT, led the researchers to determine that combining the male and female officers into one data set was appropriate, which also served to increase the sample size for the correlation analysis. Spearman’s correlations were used as they are more robust and appropriate for non-parametric data [62,63]. The correlation strength was designated as: a ρ between 0 to ±0.3 was small; ±0.31 to ±0.49, moderate; ±0.5 to ±0.69, large; ±0.7 to ±0.89, very large; and ±0.9 to ±1 near perfect for relationship prediction [64].

## 3. Results

**Figure 1:**
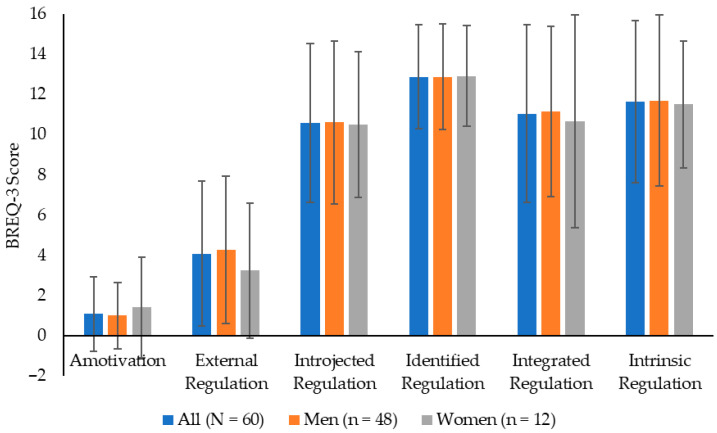
Scores (mean ± SD) for the six motivation subscales (amotivation, external regulation, introjected regulation, identified regulation, integrated regulation, and intrinsic regulation) from the 24-item Behavioral Regulation in Exercise Questionnaire (BREQ-3) completed by male and female law enforcement officers.

Figure 1 displays the mean data for each of the motivation scores. The highest mean score was for identified regulation (12.88 ± 2.57), followed by intrinsic (11.65 ± 4.03), integrated (11.05 ± 4.42), and introjected (10.59 ± 3.95) regulation. The Kolmogorov–Smirnov determined that all 6 variables were not normally distributed (*p* ≤ 0.035), so Mann–Whitney U-tests were used. There were no significant between-sex differences in any of the motivation subscales (*p* = 0.399–0.966; *d* = 0.02–0.29), with the effects from trivial-to-small.

Table 3 displays the PA and RT data, all of which were not normally distributed (*p* ≤ 0.007). Mann–Whitney U-tests were again used to derive any between-sex differences. Male officers completed significantly more moderate sessions per week, which had a moderate effect. No other PA or RT variables were significantly different between the sexes, with effects ranging from trivial-to-moderate. For the health and fitness test data (Table 4), all tests were normally distributed except for grip strength (*p* < 0.001). Thus, independent samples-*t*-tests were used to compare the sexes for all the health and fitness assessments except for grip strength, where the Mann–Whitney U-test was adopted. The male officers had significantly greater RHR, BP, SMM% (all moderate effects), WHR, grip strength (both very large effects), and push-ups (large effect). The female officers had greater FM% and sit-and-reach (both moderate effects).

Strenuous sessions per week had significant moderate relationships with introjected (ρ = 0.301, *p* = 0.020) and intrinsic (ρ = 0.448, *p* < 0.001) regulation, and large relationships with identified (ρ = 0.563, *p* < 0.001) and integrated (ρ = 0.512, *p* < 0.001) regulation (Table 5). Activity score had a small relationship with intrinsic regulation (ρ = 0.287, *p* = 0.026), and moderate relationships with identified (ρ = 0.472, *p* < 0.001) and integrated (ρ = 0.356, *p* < 0.001) regulation. RTF had a small negative relationship with amotivation (ρ = −0.295, *p* = 0.022). RTF had a positive small relationship with introjected regulation (ρ = 0.286, *p* = 0.027), moderate relationship with intrinsic regulation (ρ = 0.426, *p* < 0.001), and large relationships with identified (ρ = 0.514, *p* < 0.001) and integrated (ρ = 0.605, *p* < 0.001) regulation. RT3M had a small negative relationship with amotivation (ρ = −0.290, *p* = 0.025), a moderate positive relationship with intrinsic regulation (ρ = 0.447, *p* < 0.001), and large positive relationships with introjected (ρ = 0.503, *p* < 0.001), identified (ρ = 0.670, *p* < 0.001), and integrated (ρ = 0.585, *p* < 0.001) regulation. RT7D had positive moderate relationships with identified (ρ = 0.444, *p* < 0.001) and integrated (ρ = 0.341, *p* = 0.008) regulation.

RHR had a positive small relationship with external regulation (ρ = 0.292, *p* = 0.024; Table 6). Negative relationships were shown between RHR and introjected (ρ = −0.338, *p* = 0.008) and identified (ρ = −0.270, *p* = 0.037) regulation, which were moderate and small, respectively. SMM% had positive relationships with integrated (ρ = 0.323, *p* = 0.012; moderate) and intrinsic (ρ = 0.291, *p* = 0.024; small) regulation. FM% exhibited negative relationships with integrated (ρ = −0.318, *p* = 0.013; moderate) and intrinsic (ρ = −0.265, *p* = 0.041; small) regulation. Push-ups had positive relationships with introjected (ρ = 0.298, *p* = 0.021; small), identified (ρ = 0.398, *p* = 0.002; moderate), integrated (ρ = 0.511, *p* < 0.001; large), and intrinsic (ρ = 0.486, *p* < 0.001; moderate) regulation. Sit-ups related with identified (ρ = 0.332, *p* = 0.010; moderate), integrated (ρ = 0.430, *p* < 0.001; moderate), and intrinsic (ρ = 0.292, *p* = 0.024; small) regulation.

## 4. Discussion

This study investigated the relationships between exercise motivation with PA, RT, and health and fitness in law enforcement officers. It was hypothesized that officers who exhibited greater intrinsic motivation would be more physically active and have better health and fitness. The results provided some support to these hypotheses. Overall, intrinsic motivation appears to play an essential role in being physically active and maintaining better body composition and muscular endurance for an officer. While there could be bidirectionality of the observed relationships (i.e., better fitness may enhance motivation, and vice versa), it is still important to acknowledge the potential value in developing intrinsic motivation in officers. As will be discussed, law enforcement training staff could develop an officers’ intrinsic motivation during their time in the academy and as they start their career post-academy [65]. This is important, especially as many law enforcement training academies utilize and focus on extrinsic motivational techniques rather than intrinsic motivational techniques [36,37,38].

To provide context for the fitness of the officers, Lockie et al. [12] provided a detailed profile of the sample. To summarize some of the key findings, approximately 87% of the officers were fatter than average or above, 72% had very poor RHR, 87% had elevated blood pressure, 54% and fair-to-poor flexibility as measured by the sit-and-reach, 51% had fair-to-poor grip strength, and 80% had average-to-very poor step test recovery HR. As Lockie et al. [12] noted, these law enforcement officers, and officers in general, needed more support in maintaining or improving their health and fitness. Further, these data emphasize why it is essential to understand an officer’s motivation towards PA and RT and how it could influence health and fitness. Motivation is especially important as many law enforcement organizations do not mandate PA or fitness standards for their officers [28]. In a survey of Peace Officer Standards and Training organizations in the USA, Lockie and Dulla [28] detailed that 43 of 50 states did not mandate physical fitness standards for their incumbent officers. In many cases it is up to the individual officer to maintain their overall well-being, so understanding why they may or may not exercise would be valuable information for the practitioner.

The results from the BREQ-3 suggested that the officers generally valued the benefits of PA and exercise [41,42]. Identified regulation (personal importance/value placed on exercise) had the highest mean score, then intrinsic (exercise engagement due to personal satisfaction), integrated (exercise consistent with identity), and introjected (exercise regulated by internal rewards) regulation. The presentation of these results is important, as no current research has reported BREQ-3 data for law enforcement officers. However, the data from this study exhibited some differences to an 18-item BREQ-2 questionnaire completed by physically active Norwegian men and women from endurance sports (*N* = 1198; mean age = 49.09 ± 11.39 years), which had all motivation styles except for integrated regulation [66]. The data from Havnen et al. [66] illustrated how individuals more intrinsically motivated tend to be active. Compared to the current study, Havnen et al. [66] reported mean data for amotivation (men = 0.06, women = 0.04 vs. officers = 1.08), and external (men = 0.57, women = 0.51 vs. officers = 4.08), introjected (men = 5.41, women = 5.65 vs. officers = 10.59), identified (men = 11.22, women = 11.35 vs. officers = 12.88), and intrinsic (men = 14.49, women = 14.45 vs. officers = 11.65) regulation. As can be observed, the officers from the current study had higher scores for amotivation, external, and introjected regulation, and a lower score for intrinsic regulation. These data may be reflective of their typical training environment for officers (i.e., an emphasis on external motivation) [36,37,38]. The current motivation results are even more pertinent when considering the relationships between motivation, PA, and RT.

Introjected, identified, integrated, and intrinsic regulation positively related to the number of strenuous exercise sessions per week, RTF, and RT3M. Identified, integrated, and intrinsic regulation positively related to activity score, where strenuous exercise was weighted more heavily in the calculation [40]. Identified and integrated regulation positively related to RT7D. In contrast, amotivation negatively correlated with RTF and RT3M, which indicated a lack of intention to perform RT. The implication of these results is that some form of intrinsic motivation appears essential for completing strenuous PA and RT in law enforcement officers. To develop self-determination and intrinsic motivation to exercise, an individual should ideally have autonomy, which is ownership of one’s exercise behavior; relatedness, which is where exercise satisfies the individual’s social needs; and competence, where the individual is confident they can successfully complete the exercise program [67,68]. Although academy training could still be used to impart recruit discipline [37,38], training staff should also be cognizant of developing autonomy, relatedness, and competence in recruits [67,68]. This could have the downstream effects of ensuring officers complete strenuous PA and RT once they exit the academy and start working. The importance of this is also reflected in select health and fitness results.

Greater external regulation was associated with a higher RHR, while greater introjected and identified regulation related to a lower RHR. For officers reliant on external motivation for PA, once the source of external motivation has been removed (i.e., successful completion of the training academy), the individual may not adhere to regular PA. In contrast, those officers regulated by internal rewards or those who placed importance on exercise likely had a lower RHR. These data could be linked to the PA results; officers completing more strenuous PA could experience a lower RHR. Following endurance training, both men and women can experience a reduction of approximately 5–9% in RHR, which equates to about 3–6 bpm [69]. No previous research has investigated relationships between exercise motivation and cardiovascular variables such as RHR in law enforcement officers, which makes drawing comparisons across the literature challenging. However, this is also what makes the provision of the current findings essential for the context of law enforcement training, especially as a lower RHR has been associated with better fitness [27] and reduced risk of cardiovascular disease [70]. Further, for each 10 bpm increase in RHR, there is an increase in relative risk of 9% for sudden cardiac death, 15% for cardiovascular disease, and 17% for all-cause mortality [71]. If exercise motivation could contribute to lowering RHR, this would be critical for the well-being of law enforcement officers.

Integrated and intrinsic regulation had positive relationships with SMM%, and negative relationships with FM%. That is, officers for whom exercise was part of their identity, and those who enjoyed completing exercise, had better body composition profiles shown by greater SMM% and lower BF%. Intrinsically motivated officers may complete more strenuous PA and RT. and have better body composition profiles as a result. Previous law enforcement research has established links between body composition and fitness in law enforcement personnel [48,51,61]. To provide a specific example in recruits, Collins et al. [61] detailed different measures of SMM (measured in kg) positively related to vertical jump power and medicine ball throw distance (*r* = 0.494–0.558, *p* < 0.001). Collins et al. [61] also found greater values for different FM measures (also measured in kg) related to poorer vertical jump, 75-yard pursuit run, push-ups, sit-ups, and multistage fitness test performance (*r* = ±0.371–0.557, *p* < 0.001). Additionally, a lack of PA has been linked with greater body adiposity in adults [72,73]. The current results further emphasize the importance of intrinsic exercise motivation development in law enforcement officers.

Motivation also had relationships with muscular endurance, which was measured by the 60 s push-up and sit-up tests. Greater introjected, identified, integrated, and intrinsic regulation related to more push-up repetitions, while greater identified, integrated, and intrinsic regulation related to more sit-up repetitions. Push-ups and sit-ups are commonly used in law enforcement testing to measure muscular endurance [12,27,43,51,57,61], and these exercises can be a staple in training academies [37]. As the officers were likely very familiar with push-ups and sit-ups, they may exhibit higher motivation to train fitness components (i.e., muscular endurance) they are familiar with. However, this could come at the detriment of other training foci important for law enforcement, such as explosiveness [34], which could lead to a deficit in these areas. Training staff for recruits and officers should aim to coach and educate their personnel in different training practices (e.g., strength and power training) that ultimately benefit the health and fitness of personnel [67,68]. Another strategy that could be adopted in training for law enforcement personnel is to allow them to have input into their own programs, under the guidance of training staff [26]. A law enforcement training academy used this approach in the final weeks, with the goal being to educate recruits as to the importance of fitness and to provide them with tools for exercise program design [26]. This approach could develop exercise autonomy, relatedness, and competence in their personnel.

Nonetheless, BP, WHR, sit-and-reach, grip strength, and YMCA step test recovery HR did not demonstrate any significant relationships with motivation. It is possible that these variables may be less sensitive to motivational variation or require different assessment contexts (e.g., dynamic strength tests such as the bench press, back squat, or deadlift instead of grip strength). Further, the strength of all significant relationships ranged from small to large. These data suggest that motivation is only one part of a complex set of factors that could influence the PA, health, and fitness of law enforcement officers. Exercise self-efficacy, which is the level to which someone can start and maintain participation in exercise [20], was not included in the current research and should be investigated in future studies. Factors such as accessibility to fitness facilities, mental health, and/or diet were not taken into account when examining the role of motivation in performing regular exercise among this population [74]. Without analysis of these factors, there may be a sense of nuance and context missing from what contributes to one’s motivation to exercise, and this could have influenced the correlation analyses.

There are other limitations to consider in this study. One limitation is the sample being specific to one region of California, with a relatively small sample size (*N* = 60). Fitness standards and requirements can also vary across the country [28]. This may limit generalizability of the results to other law enforcement populations. Thus, there is a need for future studies with broader regional or national samples. Given fitness differences between men and women [10,60], which was also reflected in this study, future research could also consider detailed investigation of potential sex differences in motivation for law enforcement officers. The staff at this law enforcement agency used the BREQ-3 to measure motivation [41,42,47]. The responses to questionnaires like the BREQ-3 are subjective, which could affect the results. Moreover, other exercise motivation questionnaires could produce different findings [75]. Although a valid measure of upper-body strength, grip strength was the only strength test used by the department. Although there are challenges with implementing repetition-maximum strength testing (e.g., time and equipment constraints) [37], it would be of interest to see relationships between motivation, RT, and other strength tests (e.g., bench press, back squat, deadlift). As stated, access to facilities was not considered in this study, and that can be a major impediment to an officer’s PA [39]. Other factors such as shift work, diet, mental health, and stress were not considered in this study [15], and their impacts on motivation, PA, and health and fitness should be analyzed in future studies. The career stage of the officer could also affect their motivation to exercise, especially if their job demands change (i.e., more supervisory, sedentary work [16,76]). The current research was also cross-sectional in nature. Future longitudinal studies are needed to determine whether motivation for PA and RT can be affected by academy training strategies, and the resulting long-term implications on the health and fitness of officers.

## 5. Conclusions

To conclude, intrinsically motivated officers likely completed strenuous exercise and RT, and had better RHR, body composition, and muscular endurance. While there may not be a one-size-fits-all approach to motivating officers to engage in regular physical activity, an approach that focuses on developing different intrinsic regulatory styles with a view towards self-determination should be considered when working with law enforcement personnel. This approach could aid in fostering autonomy, relatedness, and competence in officers, and ultimately intrinsic motivation towards participating in, and adhering to, physical activity long after graduating from the academy. Longitudinal or intervention-based research studies are required to confirm whether development of intrinsic motivation in officers leads to long-term PA participation and better health and fitness outcomes.

## Figures and Tables

**Table 1:** Physical activity and resistance training questionnaire.

Considering a **7-Day period** (a week), how many times on average do you do the following kinds of physical activity for **more than 15 min** during your **free time**.	
	**Times per week**
(a) **STRENUOUS EXERCISE (HEART BEATS RAPIDLY)**	_________________
(i.e., running, jogging, hockey, football, soccer, squash, basketball, cross-country skiing, martial arts, roller skating, vigorous swimming, vigorous long-distance bicycling, boxing, Zumba, spin classes)	
(b) **MODERATE EXERCISE (NOT EXHAUSTING)**	_________________
(i.e., fast walking, baseball, tennis, easy bicycling, volleyball, badminton, easy swimming, skiing, dancing)	
(c) **MILD EXERCISE (MINIMAL EFFORT)**	_________________
(i.e., yoga, archery, fishing, bowling, horseshoes, golf, easy walking)	
The next section focuses on resistance training: a form of physical activity that is designed to improve muscular fitness by exercising a muscle or a muscle group against external resistance. Examples may include: • Performing calisthenics such as push-ups and sit-ups • Lifting weights such as dumbbells or barbells • Using resistance bands • Using weight machines	
In the past **3 months only**, how often did you engage in resistance training?	
1 2 3 4 5 6 7 Never Often	
In the past **3 months only**, what is the average number of days per week that you engaged in resistance training?	
1 2 3 4 5 6 7	
In the past **week only**, how many days did you engage in resistance training?	
1 2 3 4 5 6 7	

**Table 2:** Behavioral Regulation in Exercise Questionnaire (BREQ-3).

***WHY DO YOU ENGAGE IN EXERCISE?***					
We are interested in the reasons underlying peoples’ decisions to engage or not engage in physical exercise. Using the scale below, indicate to what extent each of the following items is true for you. Please note there are no right or wrong answers, no trick questions, and your answers will not influence your current position. We simply want to know how you personally feel about exercise.					
	Not true for me		Sometimes true for me		Very true for me
1. It’s important to me to exercise regularly	0	1	2	3	4
2. I don’t see why I should have to exercise	0	1	2	3	4
3. I exercise because it’s fun	0	1	2	3	4
4. I feel guilty when I don’t exercise	0	1	2	3	4
5. I exercise because it is consistent with my life goals	0	1	2	3	4
6. I exercise because other people say I should	0	1	2	3	4
7. I value the benfits of exercise	0	1	2	3	4
8. I can’t see why I should bother exercising	0	1	2	3	4
9. I enjoy my exercise sessions	0	1	2	3	4
10. I feel ashamed when I miss an exercise session	0	1	2	3	4
11. I consider exercise part of my identity	0	1	2	3	4
12. I take part in exercise because my friends/family/ partner say I should	0	1	2	3	4
13. I think it is important to make the effort to exercise regularly	0	1	2	3	4
14. I don’t see the point in exercising	0	1	2	3	4
15. I find exercise a pleasurable activity	0	1	2	3	4
16. I feel like a failure when I haven’t exercised in a while	0	1	2	3	4
17. I consider exercise a fundamental part of who I am	0	1	2	3	4
18. I exercise because others will not be pleased with me if I don’t	0	1	2	3	4
19. I get restless if I don’t exercise regularly	0	1	2	3	4
20. I think exercising is a waste of time	0	1	2	3	4
21. I get pleasure and satisfaction from participating in exercise	0	1	2	3	4
22. I would feel bad about myself if I was not making time to exercise	0	1	2	3	4
23. I consider exercise consistent with my values	0	1	2	3	4
24. I feel under pressure from my friends/family to exercise	0	1	2	3	4

**Table 3:** Male and female law enforcement officer (*N* = 60) descriptive data (mean ± SD) for number of strenuous, moderate, and mild intensity exercise sessions per week, overall activity score, how frequently the officer engaged in resistance training in the past 3 months (RTF), the average number of days resistance training per week in the past 3 months (RT3M), and the number of days in the past week the officer engaged in resistance training (RT7D).

Physical Activity and Resistance Training Variables	All (*N* = 60)	Men (*n* = 48)	Women (*n* = 12)	*p*	*d*
Strenuous Sessions (weekly session number)	2.39 ± 1.61	2.50 ± 1.64	1.96 ± 1.48	0.367	0.34
Moderate Sessions (weekly session number)	2.22 ± 1.83	2.47 ± 1.88	1.21 ± 1.23 *	0.034	0.71
Mild Sessions (weekly session number)	2.44 ± 2.29	2.65 ± 2.40	1.63 ± 1.58	0.260	0.45
Activity Score	39.93 ± 22.34	42.78 ± 23.02	28.54 ± 15.33	0.056	0.65
RTF (scale from 0–7)	4.30 ± 1.93	4.40 ± 1.83	3.92 ± 2.35	0.482	0.25
RT3M (session number from past 3 months)	3.06 ± 2.00	3.14 ±1.97	2.75 ± 2.18	0.627	0.19
RT7D (weekly session number)	1.63 ± 1.70	1.73 ± 1.82	1.25 ± 1.06	0.621	0.28

* Significantly (*p* < 0.05) different from the male officers.

**Table 4:** Law enforcement officer (*N* = 60) descriptive data (mean ± SD) for resting heart rate, systolic and diastolic blood pressure, skeletal muscle mass [SMM%] and fat mass [FM%] percentage, waist-to-hip ratio, sit-and-reach, combined grip strength from both hands, 60 s push-ups, 60 s sit-ups, and recovery heart rate following the YMCA step test.

Health and Fitness Variables	All (*N* = 60)	Men (*n* = 48)	Women (*n* = 12)	*p*	*d*
Resting Heart Rate (bpm)	89.15 ± 13.02	91.40 ± 13.26	80.17 ± 6.97 *	<0.001	0.91
Systolic Blood Pressure (mmHg)	131.70 ± 16.10	134.96 ± 14.27	118.67 ± 16.98 *	0.001	1.10
Diastolic Blood Pressure (mmHg)	86.50 ± 10.24	87.90 ± 9.86	80.92 ± 10.23 *	0.033	0.70
SMM%	33.48 ± 5.81	34.75 ± 5.46	28.38 ± 4.27 *	<0.001	1.21
FM%	28.80 ± 6.76	27.12 ± 5.77	35.55 ± 6.39 *	<0.001	1.43
Waist-to-Hip Ratio	0.87 ± 0.07	0.89 ± 0.05	0.77 ± 0.04 *	<0.001	2.31
Sit-and-Reach (cm)	27.51 ± 7.61	26.19 ± 7.44	32.67 ± 6.12 *	0.007	0.90
Grip Strength (kg) §	86.74 ± 19.29	94.31 ± 12.58	57.08 ± 9.79 *	<0.001	3.08
60 s Push-ups (repetitions)	38.83 ± 15.01	42.10 ± 13.78	25.75 ± 12.82 *	<0.001	1.20
60 s Sit-ups (repetitions)	31.92 ± 8.90	32.88 ± 9.03	28.08 ± 7.51	0.096	0.55
YMCA Step Test Recovery Heart Rate (bpm)	120.80 ± 12.55	122.02 ± 12.91	115.92 ± 10.02	0.133	0.49

* Significantly (*p* < 0.05) different from the male officers. § Mann–Whitney U Test was used to compare the groups for this variable.

**Table 5:** Spearman’s correlations (ρ) between motivation subscales (amotivation, external regulation, introjected regulation, identified regulation, integrated regulation, and intrinsic regulation) from the 24-item Behavioral Regulation in Exercise Questionnaire (BREQ-3) with physical activity (weekly strenuous, moderate, and mild exercise sessions, and activity score) and resistance training (RT frequency [RTF], weekly sessions over 3 months [RT3M], and sessions in past 7 days [RT7D]).

		Amotivation	External Regulation	Introjected Regulation	Identified Regulation	Integrated Regulation	Intrinsic Regulation
Strenuous Sessions	ρ	−0.175	−0.027	0.301 *	0.563 *	0.512 *	0.448 *
	*p*	0.182	0.840	0.020	<0.001	<0.001	<0.001
Moderate Sessions	ρ	−0.039	0.062	−0.027	0.137	0.021	−0.006
	*p*	0.770	0.636	0.835	0.292	0.873	0.965
Mild Sessions	ρ	−0.017	−0.020	−0.050	−0.023	−0.135	−0.098
	*p*	0.897	0.880	0.704	0.862	0.305	0.457
Activity Score	ρ	−0.135	−0.032	0.241	0.472 *	0.365 *	0.287 *
	*p*	0.304	0.809	0.064	<0.001	0.004	0.026
RTF	ρ	−0.295 *	−0.077	0.286 *	0.514 *	0.605 *	0.426 *
	*p*	0.022	0.558	0.027	<0.001	<0.001	<0.001
RT3M	ρ	−0.290 *	0.037	0.503 *	0.670 *	0.585 *	0.447 *
	*p*	0.025	0.776	<0.001	<0.001	<0.001	<0.001
RT7D	ρ	−0.116	0.020	0.101	0.444 *	0.341 *	0.235
	*p*	0.376	0.881	0.442	<0.001	0.008	0.071

* Significant (*p* < 0.05) relationship between the two variables.

**Table 6:** Spearman’s correlations (ρ) between motivation subscales (amotivation, external regulation, introjected regulation, identified regulation, integrated regulation, and intrinsic regulation) from the 24-item Behavioral Regulation in Exercise Questionnaire (BREQ-3), with resting heart rate, systolic and diastolic blood pressure, skeletal muscle mass (SMM%) and fat mass (FM%) percentage, waist-to-hip ratio, sit-and-reach, combined grip strength, 60 s push-ups, 60 s sit-ups, and recovery heart rate (HR) from the YMCA step test.

		Amotivation	External Regulation	Introjected Regulation	Identified Regulation	Integrated Regulation	Intrinsic Regulation
Resting Heart Rate	ρ	0.222	0.292 *	−0.338 *	−0.270 *	−0.198	−0.071
	*p*	0.089	0.024	0.008	0.037	0.129	0.589
Systolic Blood Pressure	ρ	0.083	0.172	−0.012	−0.119	−0.026	0.174
	*p*	0.526	0.190	0.925	0.364	0.841	0.185
Diastolic Blood Pressure	ρ	0.055	0.006	−0.186	−0.205	−0.141	0.056
	*p*	0.677	0.967	0.154	0.116	0.282	0.668
SMM%	ρ	−0.104	−0.159	0.206	0.216	0.323 *	0.291 *
	*p*	0.428	0.225	0.114	0.098	0.012	0.024
FM%	ρ	0.077	0.102	−0.142	−0.191	−0.318 *	−0.265 *
	*p*	0.557	0.439	0.280	0.144	0.013	0.041
Waist-to-Hip Ratio	ρ	0.036	0.067	−0.042	−0.073	−0.169	−0.010
	*p*	0.785	0.610	0.750	0.580	0.196	0.938
Sit-and-Reach	ρ	0.083	0.006	−0.024	−0.057	0.014	−0.171
	*p*	0.533	0.966	0.859	0.688	0.917	0.195
Combined Grip Strength	ρ	0.005	0.005	−0.011	−0.196	0.025	−0.082
	*p*	0.968	0.968	0.932	0.137	0.851	0.539
60 s Push-ups	ρ	−0.119	−0.034	0.298 *	0.398 *	0.511 *	0.486 *
	*p*	0.365	0.798	0.021	0.002	<0.001	<0.001
60 s Sit-ups	ρ	−0.206	−0.082	0.155	0.332 *	0.430 *	0.292 *
	*p*	0.114	0.533	0.236	0.010	<0.001	0.024
YMCA Step Test Recovery HR	ρ	−0.119	−0.119	0.057	−0.109	−0.061	−0.118
	*p*	0.367	0.367	0.665	0.408	0.644	0.368

* Significant (*p* < 0.05) relationship between the two variables.

## Data Availability

Restrictions apply to the availability of these data due to ethical, legal and privacy concerns.

## Abbreviations

The following abbreviations are used in this manuscript:

ACSM	American College of Sports Medicine
s	Second
PA	Physical activity
RT	Resistance training
m	Meter
kg	Kilogram
RHR	Resting heart rate
BP	Blood pressure
YMCA	Young Men’s Christian Association
RTF	Resistance training frequency
RT3M	Average number of days resistance training per week in the past 3 months
RT7D	Number of sessions in the past 7 days the officer engaged in resistance training
BREQ-3	Behavioral Regulation in Exercise Questionnaire
bpm	Beats per minute
mmHg	milliliters of mercury
SMM%	Skeletal muscle mass percentage
FM%	Fat mass percentage
cm	Centimeters
WHR	Waist-to-hip ratio
HR	Heart rate
SD	Standard deviation
ρ	Spearman’s correlation coefficient
*p*	Significance
